# Dispersion and Stabilization of Exfoliated Graphene in Ionic Liquids

**DOI:** 10.3389/fchem.2019.00223

**Published:** 2019-04-16

**Authors:** Emilie Bordes, Bishoy Morcos, David Bourgogne, Jean-Michel Andanson, Pierre-Olivier Bussière, Catherine C. Santini, Anass Benayad, Margarida Costa Gomes, Agílio A. H. Pádua

**Affiliations:** ^1^Centre National de la Recherche Scientifique, SIGMA Clermont, Institut de Chimie de Clermont-Ferrand, Université Clermont Auvergne, Clermont-Ferrand, France; ^2^UMR 5265 Centre National de la Recherche Scientifique, Université de Lyon, Villeurbanne, France; ^3^Chemistry Department, Faculty of Science, Alexandria University, Alexandria, Egypt; ^4^Université Grenoble Alpes and CEA, LITEN, Grenoble, France; ^5^École Normale Supérieure de Lyon, Centre National de la Recherche Scientifique, Laboratoire de Chimie, Lyon, France

**Keywords:** exfoliation, graphene, graphite, ionic liquids, suspension

## Abstract

The liquid-phase exfoliation of graphite is one of the most promising methods to increase production and commercial availability of graphene. Because ionic liquids can be easily obtained with chosen molecular structures and tuneable physicochemical properties, they can be use as media to optimize the exfoliation of graphite. The understanding of the interactions involved between graphite and various chemical functions in the solvent ions will be helpful to find liquids capable of dissociating and stabilizing important quantities of large graphene layers. After a step of sonication, as a mechanical precursor, samples of suspended exfoliated graphene in different ionic liquids have been characterized experimentally in terms of flake size, number of layers, total concentration and purity of the exfoliated material. Nine different ionic liquids based on imidazolium, pyrrolidinium and ammonium cations and on bis(trifluoromethylsulfonyl)imide, triflate, dicyanamide, tricyanomethanide, and methyl sulfate anions have been tested. UV-vis, Raman and X-ray photoelectron in addition to high resolution transmission electron and atomic force microscopy have been selected to characterize suspended exfoliated graphene in ionic liquids. The number of layers in the flakes exfoliated, the size and concentration depend of the structure of the ionic liquid selected. In order to obtain large flake sizes, ionic liquids with bis(trifluoromethylsulfonyl)imide anions and a cation with an alkyl chain of medium length should be selected. Smaller cation and anion favors the exfoliation of graphene. The exfoliation caused the formation of C-H bonds and the oxidation of the graphitic surface.

## 1. Introduction

Since its isolation in 2004 (Novoselov, 2004), graphene has showed superior mechanical (Lee et al., 2008), electrical (Mayorov et al., 2011), and thermal (Mak et al., 2010) properties (Novoselov et al., 2012). Graphene is the first 2D-material available to us. Its remarquable properties make it useful in many applications in diverse fields such as energy storage with batteries (Raccichini et al., 2014), electronics with transistors (Kelly et al., 2017), photonics with photodetectors (Blake et al., 2008), coating with composites (Young et al., 2012) or biomedical with drug delivery (Nair et al., 1993). Graphite is a stack of graphene sheets bound by van der Waals interactions, each graphene layer being made of sp^2^ carbons distributed in a hexagonal crystal structure. Today, two approaches are known to produce graphene: in “bottom-up” methods, such as chemical vapor deposition (CVD), graphene is synthesized, whereas in “top-down” methods, for example liquid-phase exfoliation, graphene sheets are separated from bulk material. The first approach produces low quantities with high quality and large flakes. The second method (top-down) using graphite is low in cost and yields a high concentration of suspended flakes but fabricates limited-size sheets with a low yield of mono-layer graphene. The development of technologies to produce large quantities of high-quality exfoliated graphene is important. One way to achieve a large scalable production is to improve methods of the liquid-phase exfoliation of graphite.

Direct exfoliation of graphite in liquid media using a mechanical precursor is a promising approach in order to increase the production and the commercial availability of high-quality graphene. Various techniques (ultrasound Hernandez et al., 2008, ball-milling Zhao et al., 2010, microwaves Wang et al., 2011) and solvents such organic solvents (Hernandez et al., 2008), water/surfactants (Lotya et al., 2009), supercritical fluids Pu et al., 2009, or ionic liquids (ILs) Wang et al., 2010 have been studied in order to produce a large graphene sheets in high quality and concentration. To determine the best solvents to overcome the van der Walls interactions between two graphene layers, the surface tension of different liquids has been investigated. Hernandez et al. (2008) and Coleman (2013) have studied forty molecular solvents as media to exfoliate graphite *via* sonication and characterized them by the fraction of graphite/graphene remaining after centrifugation. They observed that solvents with a high ability to disperse graphite are the ones having a surface tensions between 40 and 50 mNm^-1^. N-methyl-2-pyrrolidone (NMP) has stabilized a high concentration of graphene flakes compared to other solvents (Coleman, 2013). However some solvents with a surface tension in the 40–50 mN m^-1^ range do not necessary achieve high level of exfoliation. Interfacial energies graphite-solvent are thus not the only descriptor to predict a good candidate for exfoliation of graphite. ILs have demonstrated efficiency to disperse graphene derivatives such as carbon nanotubes (Chen et al., 2007; Fukushima and Aida, 2007; Wang et al., 2008; Shim and Kim, 2009; Peng et al., 2013) or fullerene (Szala-Bilnik et al., 2016). The electrostatic interactions present in the ILs would be responsible for the solvating results of the graphitic planes through favorable interactions between ions and the polarisable electrons in graphene, in particular cation-π interactions.

Ionic liquids (ILs) are liquid salts below 373 K (Seddon, 1997; Hallett and Welton, 2011), generally constituted of a bulky organic cation with an inorganic or organic anion. ILs can be easily obtained with chosen molecular structures in function of the intended application. Their very low vapor pressure makes the ILs less hazardous (reduction of flammability) than organic solvents in general. Among the most representative properties are their ionicity (Hollóczki et al., 2014), their chemical and thermal stability, their large electrochemical window (Armand et al., 2009) and ease of recycling. A large number of ion combinations is possible, leading to a wide range of physicochemical properties (Deetlefs et al., 2006). Inks (Kelly et al., 2017), supercapacitors (Chen et al., 2011, 2012; Tsai et al., 2013; Li et al., 2016; Liu et al., 2017), filter sponges (Zambare et al., 2017), graphene-polymer composites (Peng et al., 2013; Saurín et al., 2016; Wang et al., 2017) belong to the list where graphene and graphite meet ionic liquids for application purposes.

The direct exfoliation of graphite (without an oxydation step) has been reported in ILs with promising results. In 2010, Wang et al. (2010) were the first group to study the exfoliation of graphite using two ILs; 1-butyl-3-methylimidazolium bis(trifluoromethanesulfonyl)imide ([C_4_C_1_im][Ntf_2_]) and 1-butyl-1-methylpyrrolidinium bis(trifluoromethanesulfonyl)imide ([Pyrr_4, 1_][Ntf_2_]). The exfoliation conditions are summed up in Table 1. According to the results obtained by X-ray photoelectron spectroscopy (XPS), the exfoliation did not lead to any major oxidation to graphene flakes. In the same year, 1-hexyl-3-methyl-imidazolium hexafluorophosphate ([C_6_C_1_im][PF_6_]) was also used to exfoliate graphite using an ultrasound technique (Nuvoli et al., 2011). The authors demonstrated that a longer sonication time and a high initial concentration are favorable conditions to achieve higher concentrations of exfoliated graphene.

**Table 1:** Comparison of ILs used for the direct exfoliation of graphite in the liquid phase.

**ILs**	**Exfoliation set up**	**Yield**	**Size**	**Nbr. layer**
[C_4_C_1_im][Ntf_2_] (Wang et al., 2010)	Ultrasound(750 W)/1 h + 10,000 rpm/1 h	47.5%	≈ μm	1–5
[Pyrr_4, 1_][Ntf_2_] (Wang et al., 2010)	”	”	”	”
[C_6_C_1_im][PF_6_] (Nuvoli et al., 2011)	Ultrasound(550 W)/24 h + 4,000 rpm/0.33 h	8.17%	3-4 μm	1–5
[C_4_C_1_im][Ntf_2_] (Bari et al., 2014)	Ultrasound(10 W)/1 h + 5,000 rpm/6 h	unstable	n/a	n/a
[BnzC_1_im][Ntf_2_] (Bari et al., 2014)	”	0.81%	n/a	n/a
[(Bnz)_2_im][Ntf_2_] (Bari et al., 2014)	”	58%	n/a	2-5
[C_4_C_1_im][PF_6_] (Shang et al., 2012)	Grinding/4 h + 3,000 rpm/0.5 h	20%	9 nm	2-7
[(ethoxy)im][PF_6_] (Matsumoto et al., 2015)	Microwaves(30 W)/0.5 h	92%	1–5 μm	1

*Description of the methods used to produce exfoliated graphene, mass yield, lateral size of the flakes and number of exfoliated layers. (“n/a” means non available information)*.

New ILs have been synthesized for the specific needs of graphene exfoliation. The approach of Bari et al. (2014) was to functionalise an imidazolium cation with one or two benzyl groups in order to create π-π interactions in contact with graphenic planes. Ultrasound was used to supply energy for exfoliation of graphite into suspensions. Four different ILs were investigated: 1-benzyl-3-methylimidazolium bis(trifluoromethylsulfonyl)imide ([BnzC_1_im][Ntf_2_]), [C_4_C_1_im][Ntf_2_], 1-benzyl-3-metylimidazolium bromide ([BnzC_1_im][Br]) and 1,3-bis(phenylmethyl)imidazolium bis(trifluoromethylsulfonyl)imide ([(Bnz)_2_im][Ntf_2_]). The concentration of graphene in suspension was measured by UV-vis absorbance spectroscopy. [BnzC_1_im][Br] has a viscosity that was judged too high to disperse graphite. The concentration of suspended graphene in the [C_4_C_1_im][Ntf_2_] was too low to be detected by absorbance with the author's set up. [(Bnz)_2_im][Ntf_2_] stabilized a larger amount of graphene over [BnzC_1_im][Ntf_2_] (values are listed in Table 1). This results were explained by *ab initio* calculations, as both benzyl groups of [(Bnz)_2_im][Ntf_2_] were found oriented parallel to the surface of graphene unlike the single benzyl substituent in [BnzC_1_im][Ntf_2_] (Bari et al., 2014). No statistical study was actually carried out to characterize this heterogeneous suspension, either in terms of lateral size of the flakes or the number of layers of exfoliated graphene. A better understanding of the efficacy of ILs to exfoliate graphene is needed.

In 2015, Matsumoto et al. (2015) developed a fast and effective method to exfoliate natural graphite. A 25 mg mL^−1^ suspension of graphite powder in an oligomeric IL with repeating units of [(ethoxy)im][PF_6_], was exfoliated using microwaves during 0.5 h. The mixture reached the temperature of 443 K. By proton NMR the authors showed that 10% of the IL was degraded. Graphene had no additional structural defects and 95% of the flakes produced were single-layer graphene. The same protocol was used in [C_4_C_1_im][PF_6_] but with a lower exfoliation rate.

In 2016, Elbourne et al. (2016) explored the spontaneous exfoliation of a highly oriented pyrolytic graphite (HOPG) immersed into five ILs without mixing or sonication. Two of the 5 ILs studied, [C_2_C_1_im][Ntf_2_] and [C_2_C_1_im][acetate], degraded the surface of the HOPG and exfoliated graphene layers after 72 and 190 min, respectively. To confirm these observations, atomic force microscopic (AFM) was used to analyse the surface showing that the IL intercalated between graphene layers and that the cation preferentially interacts with the surface of graphene compared to the anion. One explanation is that the C_2_C_1_im^+^ cation is not very large and can be interposed between two layers of graphene. Also the interfacial energy between the IL and graphene is comparable to that between two stacked graphene sheets.

It is not possible to compare two solvents without using the same exfoliation conditions. In this study we compared the graphite exfoliation in nine different commercial ILs in order to establish structure/property rules for the exfoliation process. We make use of a number of characterization techniques to assess the concentration of suspended graphene, flake size, number of layers, and chemical integrity of the exfoliated material.

## 2. Methods

### 2.1. Materials

The ionic liquids (ILs) studied in this work are listed in Table 2. All are liquids at room temperature. The ILs were dried under primary vacuum for 24 h at room temperature. The water content was determined with a Karl Fisher coulometer DL32 from Metter Toledo in a Hydranal solution and is listed in Table S1.

**Table 2:** Structure, name and origin of the ILs selected for the liquid phase exfoliation of graphite.

**Structure**		**Abbreviation**	**Origin**
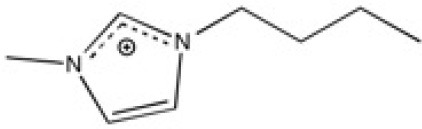	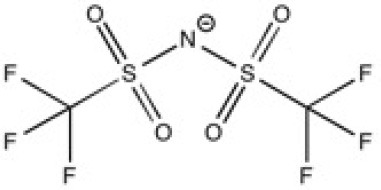	[C_4_C_1_im][Ntf_2_]	Iolitec (99 %)
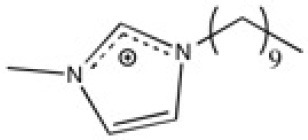	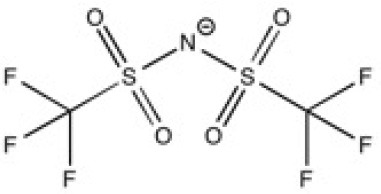	[C_10_C_1_im][Ntf_2_]	Iolitec (98%)
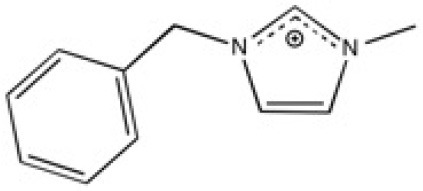	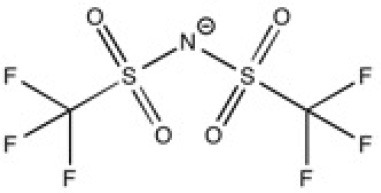	[BnzmC_1_im][Ntf_2_]	Iolitec (99%)
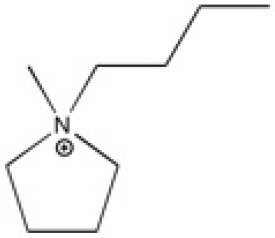	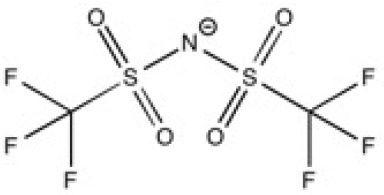	[Pyrr_4, 1_][Ntf_2_]	Iolitec (99%)
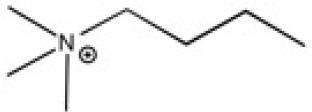	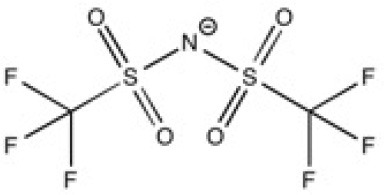	[N_4, 1, 1, 1_][Ntf_2_]	Iolitec (99%)
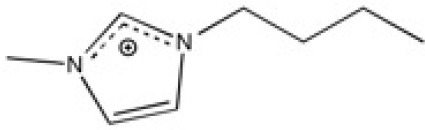	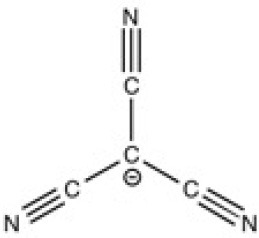	[C_4_C_1_im][C(CN)_3_]	Iolitec (98%)
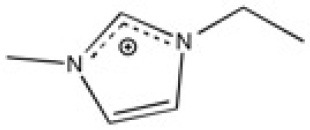	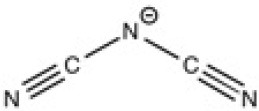	[C_2_C_1_im][N(CN)_2_]	Iolitec (98%)
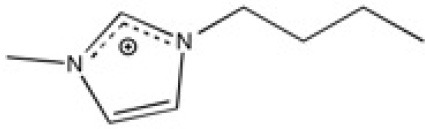	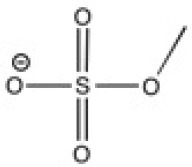	[C_4_C_1_im][C_1_SO_4_]	Iolitec (99%)
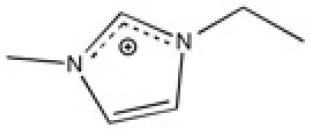	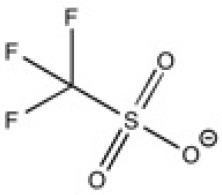	[C_2_C_1_im][Otf]	Iolitec (99%)

Natural graphite flakes were purchased from Alpha Aesar with a 99.8% purity and a size inferior to 325 mesh. Raman spectroscopy and X-ray diffraction (XRD) were performed, without any treatment, to verify the initial purity (see in the Figure S1A).

In order not to degrade the IL and to work at low temperature with a scalable technique, ultrasound was selected as a mechanical precursor technique. The chemical stability of the IL during sonication was verified by XPS (see Figure S6). To have a significant evaluation of the stabilization of graphene in ILs, a high rate of centrifugation was employed. The concentration was estimated by UV-visible spectroscopy. The lateral size of the flakes was measured by transmission electron microscopy (TEM) and AFM. The number of layer was identified by AFM and Raman spectroscopy. The purity of the exfoliated graphene was determined by XPS.

### 2.2. Sample Preparation

Suspended exfoliated graphite (SEG) solutions were prepared by adding graphite flakes to 2 mL of IL at an initial concentration of 5 mg.mL^−1^. To produce a high concentration suspension of a few layers or monolayer graphene, these dispersions were then sonicated in a low power sonicator bath (Branson 2510, 40 kHz, 130 W) for 24 h. The ultrasonic water bath warmed up during use and reached a maximum temperature of 328 K. This thermal energy expanded the graphite layers and made the exfoliation process easier. As we worked with ILs, this temperature increase did not cause degradation of the solvent. Thus, the water bath was not cooled but was maintained at 323 ± 5 K. The horizontal distribution of the ultrasound is not homogeneous in the bath (Nascentes et al., 2001). In order to obtain reproductible results we developed a rotating holder (80 rpm) to support the samples, which eliminates the arbitrary position of the sample in the bath. For the graphene suspensions to exhibit long term stability, the dispersions were centrifuged at 10,000 rpm for 1 h at 298 K using a centrifuge (Thermoscinetic Sorval biofuge Primo R), (G-force equals to 9,727 g). After centrifugation of the supernatants or SEG, *ca*. 1.5 mL was carefully removed and retained for further use. A set of ten dispersions of graphite flakes in [C_4_C_1_im][Ntf_2_] were prepared in order to determine the repeatability of the experiment. Six different sonication experiments were conduced under the same conditions. After the centrifugation steps, the absorptions of the supernatants at 660 nm were measured. The standard deviation of those absorption values was 40%.

For the remaining ILs, two samples were prepared at the same time. We tried to exfoliate graphite flakes in 1-butyl-3-methylimidazolium bis(2-ethylhexyl)sulfosuccinate ([C_4_C_1_im][AOT]) from Brown et al. (2012), but because of its high viscosity, the flakes could not be suspended in the IL.

### 2.3. Sample Characterization

To estimate the quantities of SEG in the nine ILs, we used UV-vis spectroscopy. The absorption measurements were performed by a spectrophotometer Jasco V-650 at 660 nm (Hernandez et al., 2008; Lotya et al., 2009; Khan et al., 2010; Nuvoli et al., 2012) with a 1 cm optical path quartz cuvette at ambient temperature. The signal from the IL was systematically subtracted. Just after mixing the graphite flakes with the IL, the suspensions were shaken and the absorbances of the solutions were quickly measured to prevent any sedimentation. After the sonication and the centrifugation steps, the absorbances of SEG were determined. The ratio of the two absorbances gives us the amount of material stabilized in IL.

All samples were analyzed by high resolution transmission electron microscopy (HRTEM) using a MET JEOL 2100FEF (field effect gun energy filtering) microscope with a point resolution of 0.23 nm without removing the IL. First, a thin film of SEG in IL was deposited on a copper mesh 200 grid coated with a layer of formvar/carbon. The grid was then dried gently with a filter paper. The liquid films were observed using an acceleration voltage of 200 kV. For each sample, five squares of the grid were analyzed. High resolution transmission electron microscopy, HRTEM, analysis was also performed on some particles using the same microscope. Subsequently, the HRTEM images and their crystal structure were determined from the diffraction pattern seen in the Fourier Transform images.

A volume of 1 mL of SEG was filtered through a polyvinylidene fluoride (PVDF) hydrophobic membrane (from Durapore) with a 220 nm pore size. The membrane was washed with dichloromethane and isopropanol (IPA) to remove the IL from the exfoliated graphite. Then, the filter was dried at 463 K during 12 h. This filter with exfoliated graphite was analyzed by Raman spectroscopy. Raman spectra were recorded at 294 K on a Jobin Yvon T64000 spectrophotometer equipped with a Olympus confocal microscope (100 × objective lens) with a CDD multichannel detector cooled with liquid nitrogen. The excitation source was a 514.5 nm ionized argon laser (Spectra Physics) line. The laser power was adjusted to 200 mW (minimal power). Spectral resolution is at 0.7 cm^−1^ in the range of 1,200–2,900 cm^−1^.

The membrane filter with exfoliated graphite was washed from the opposite side with 2 mL of IPA to remove the nanomaterial. Ten drops of IPA suspension (20 μL per drop every 1 min) were casted by spin coating at 1,000 rpm onto a silicium wafer (Si/SiO_2_). The Si/SiO_2_ wafer had been previously washed in IPA during 30 min in an ultrasonic bath. After coating with the exfoliated graphene, the wafer was dried at 463 K for 2 h. The lateral and vertical size of the particules on the wafer were measured by AFM (Table S3). A minimum area of 600 μm^2^ was explored on the silicium wafer where the homogeneous deposition is visible in the Figure S3. The arithmetic mean roughness (Ra) was estimated on a area of 1,500 μm^2^ of the SiO_2_/Si wafer after receiving ten drops of IPA followed by drying for 2 h at 463 K. The Ra obtained is 0.234 nm.

AFM measurements were carried out on a Bruker Mutlimode 8 equiped with a Nanoscope 5 using Tapping Mode and Peak Force Tapping based (Bruker Nano Inc., 2017) on real time force distance curve analysis recorded at a frequency of about 2.0 kHz. This allows us to locally measure the height of the sample. In addition, the Peak Force QNM mode controls the force applied to the sample by the tip, this decreasing the contact area between the tip and sample as well as deformation depths. So there is minimal damage to the probe or sample. The analyses were performed using a RTESPA tip (300 kHz) from Bruker. The given characteristics of the RTESPA tip corresponded to a spring constant of 40 N m^-1^ and a curvature radius of 8 nm. The scan rate of the sample is 0.5 Hz with 512 scan lines.

XPS analysis was performed with Versaprobe II PHI 5000(ULVAC-PHI) spectrometer using a 100 μm focused monochromatic Al-Kα X-ray source (1486.6 eV) beam. The high-resolution spectral analysis was performed using a pass energy of 23 eV allowing an energy resolution of 0.5 eV. The XPS spectra were fitted by using Multipak V_9.1_ software in which a Shirly background is assumed. The fitting peaks of the experimental spectra are defined by a combinaison of Gaussian (80%) and Lorentzian (20%) distributions. The analyzed covered a surface of approximately 100 μm^2^ with a depth of 5 nm.

The Potentiel of Mean Force (PMF), calculated in this work, corresponds to the reversible work required to peel one layer of graphene from a stack of four layers in different ILs. The method and set up of these PMF calculations by molecular dynamics have been described in a previous paper (Bordes et al., 2018). The ionic liquids were modeled by the CL&P atomistic force field (Canongia Lopes and Pádua, 2012) and graphene/graphite by the OPLS-AA force field for aromatic carbon materials (Severance and Jorgensen, 1990). These are fully-atomistic force fields representing the covalently bonded structures with full flexibility, through harmonic covalent bonds and valence angles, and also torsion energy profiles that determine molecular conformations and the flexibility of the 2D nanomaterial. Non-bonded interactions included Lennard-Jones potentials and electrostatic partial charges placed on each atom. The periodic simulation boxes consisted of a stack of five stacked layers of graphite (each sheet about 5 by 4 nm) surrounded by at least 2 nm of ionic liquid, in such a way that the stack does not interact with its periodic images. The systems contain about 25,000 atoms. Simulations were carried out using the LAMMPS (Plimpton, 1995) molecular dynamics package, at 423 K and 1 bar using Nosé-Hoover thermostat and barostat. Long-range electrostatic interactions were computed using the PPPM method. The length of covalent bonds terminated by H atoms were constrained using the SHAKE algorithm in order to allow for a timestep of 1 fs. Trajectories of 5 ns were generated after 1 ns equilibration. The reversible work of peeling the top layer from the stack, in vacuum and in the presence of ionic liquid, was evaluated using a potential of mean force technique (umbrella sampling with the weighed histogram analysis method). The authors have used similar methods studying exfoliation of different nanomaterials in molecular and ionic solvents (Sresht et al., 2015, 2017; Bordes et al., 2018), with further simulation details provided in these previous publications.

## 3. Results and Discussion

### 3.1. Concentration of SEG in ILs

**Figure 1:**
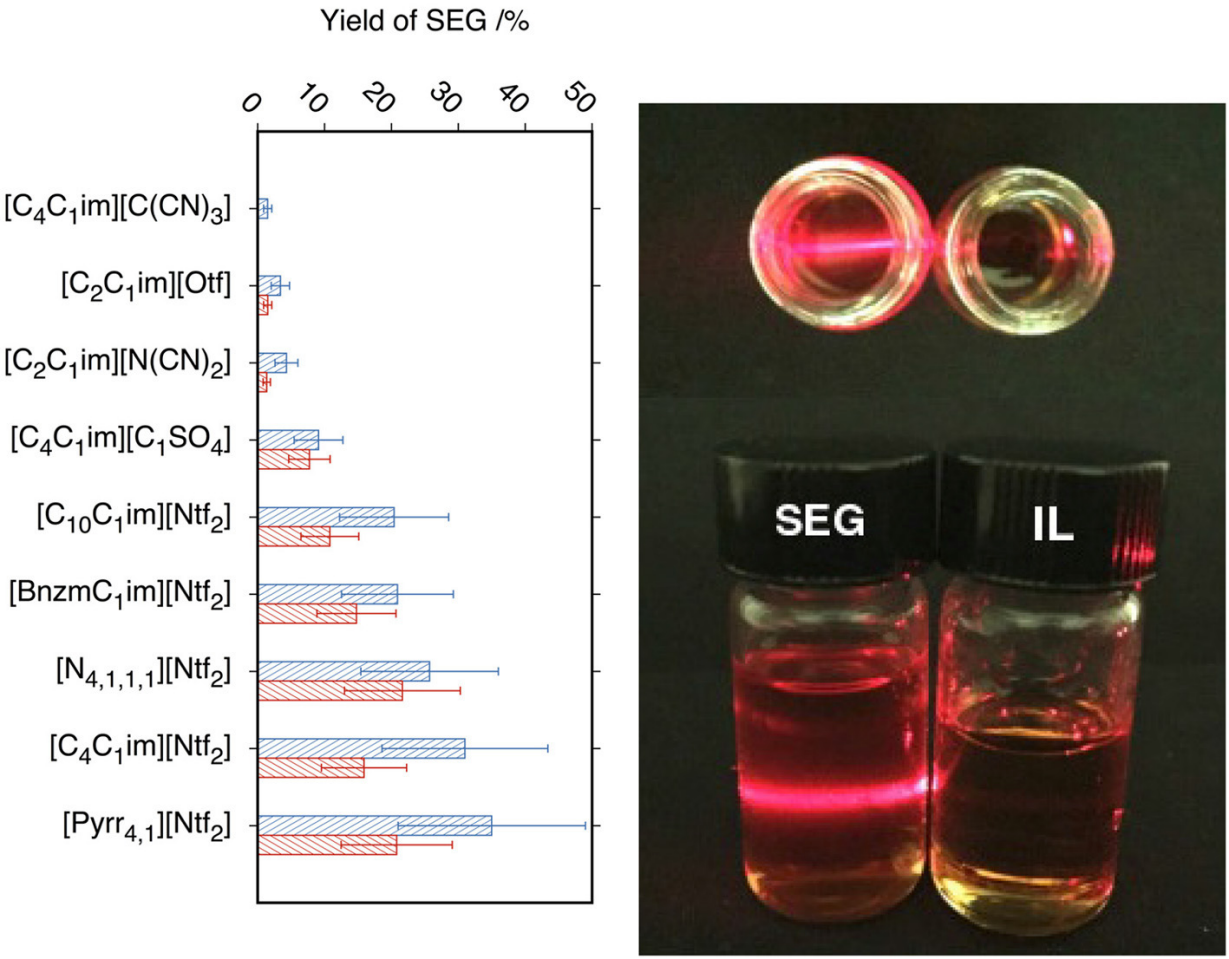
On the left, the histogram represents the percentage of remaining suspended exfoliated graphite (SEG) in ionic liquid (IL) after centrifugation (blue) and after filtration of particules with a flakes size above 220 nm (red). On the right, pictures show Tyndall effect on SEG in [C_4_C_1_im][Ntf_2_], using a red laser.

The total concentration of carbon material in suspension in ILs was estimated by absorbance spectroscopy at 660 nm. The concentration represents the surface of exfoliated graphite flakes that absorb light. In Figure 1, pictures of a SEG solution and corresponding ILs representing the Tyndall effect are presented and the scattered light in the suspension is seen. Thus, it is clear that exfoliated graphite can be dispersed in ILs.

In order to have a first assessment of the solvent ability of ILs, after the sonication and the centrifugation step, we have measured the absorbance of the supernatant suspensions containing exfoliated graphite. After filtration of the supernatant suspensions through a PVDF filter with 220 nm pores, the absorbance of filtrated suspensions were measured and subtracted to the absorbance of the supernatant suspensions. The Figure 1 represents the yields of SEG in supernatant suspension (blue histogram) and the yield of SEG above 220 nm of size (red histogram). The yield of SEG corresponds to the ratio of the absorbance of nano-carbon in ILs after exfoliation to the absorbance measured before exfoliation. Quantities of suspended graphite are given in Table S2.

The [C_4_C_1_im][Ntf_2_], [N_4, 1, 1, 1_][Ntf_2_] and the [Pyrr_4, 1_][Ntf_2_] presented the highest yield of SEG (the concentration is around 1.8 mg.mL^−1^) among the nine studied ILs. In the ILs with other anions, the concentrations are below 0.5 mg.mL^−1^ and lower than in ILs with the ${\text{Ntf}}_{2}^{-}$ anion. The [C_4_C_1_im][C(CN)_3_], [C_2_C_1_im][Otf] and [C_2_C_1_im][N(CN)_2_] ILs presented the lowest concentrations of SEG. The relatively high error bars make the distinction between ILs with the ${\text{Ntf}}_{2}^{-}$ anion but different cations difficult. Bari et al. (2014) exfoliated graphene in [BnzC_1_im][Ntf_2_] and also in [C_4_C_1_im][Ntf_2_] with a different set up. After exfoliation, no nano-carbon object was found in [C_4_C_1_im][Ntf_2_] and 0.81% of SEG in [BnzC_1_im][Ntf_2_]. The low and similar concentrations of SEG reported in these two ILs hinders comparison with our results. Our main conclusion is that using a ${\text{Ntf}}_{2}^{-}$ anion instead of other anions is favorable to stabilize exfoliated graphene.

**Figure 2:**
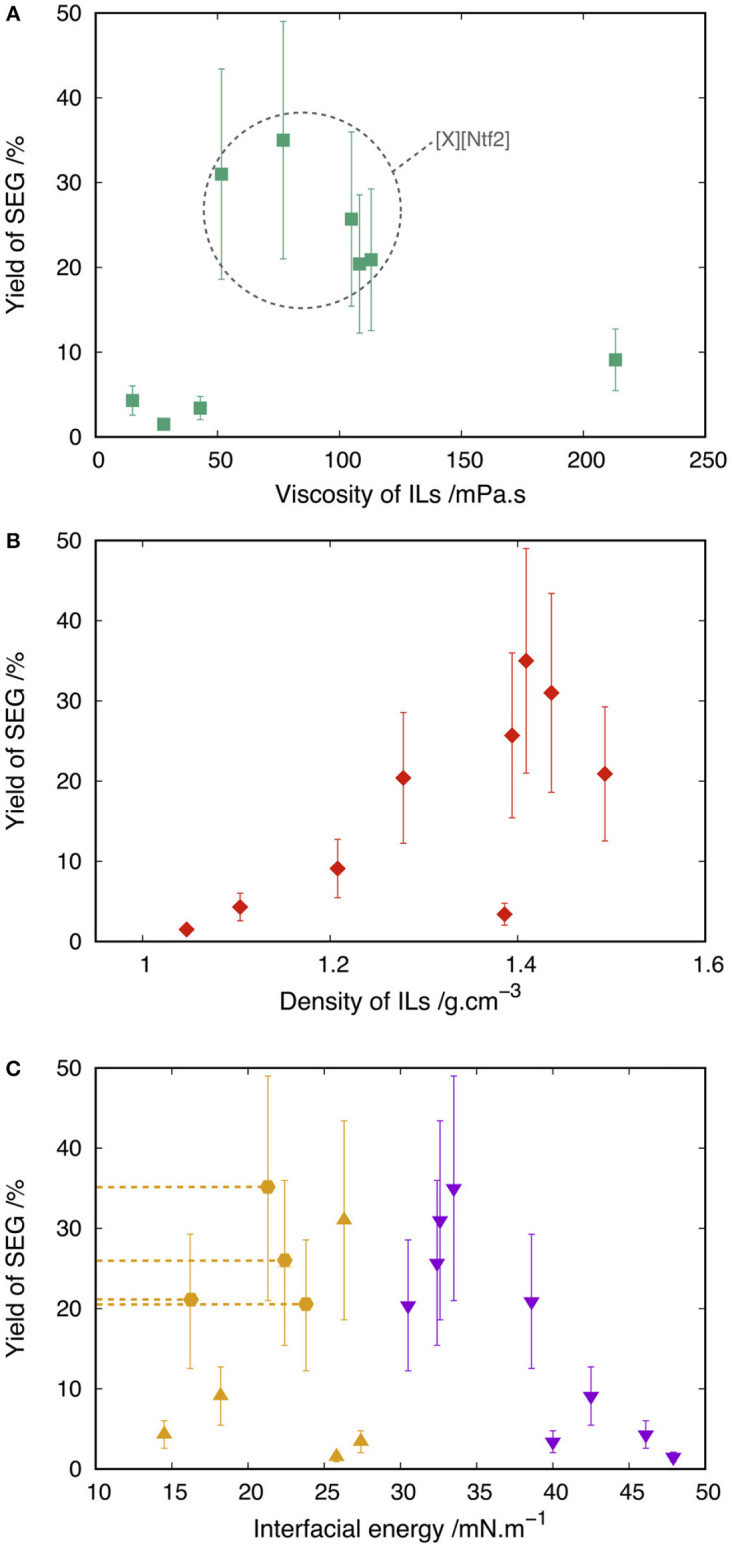
Concentration of suspended graphite in ILs as function of properties of ILs at 298 K: **(A)** viscosity of ILs (Dzyuba and Bartsch, 2002; Seddon et al., 2002; Jacquemin et al., 2006; Pereiro et al., 2007; Ahosseini and Scurto, 2008; Fröba et al., 2008; Harris et al., 2011; Almeida et al., 2016) (green squares), **(B)** density of ILs (Seddon et al., 2002; Jacquemin et al., 2006; Pereiro et al., 2007; Fröba et al., 2008; Kolbeck et al., 2010; Tao et al., 2014; Almeida et al., 2016) (red diamonds), **(C)** ILs-argon surface tension (Bordes et al., 2017) (purple triangles), graphite-ILs interfacial energy (Bordes et al., 2017) (orange triangles) and upper bound to the graphite-ILs interfacial energy (orange hexagons) (Bordes et al., 2017).

In Figure 2 is presented the yield of SEG as function of the viscosity, density, surface tension of the studied ILs and of the interfacial energy between graphite and the ILs. The concentration of SEG in ILs is not correlated to their viscosity. For example, [C_4_C_1_im][C_1_SO_4_] which is the IL with the highest viscosity, the concentration of SEG is lower than in the ILs based on the ${\text{Ntf}}_{2}^{-}$anion which have lower viscosities. These results validate the experimental method followed and prove that the chosen rate of centrifugation is sufficiently high to balance the IL viscosity during the stabilization of the SEG. The ILs based on ${\text{Ntf}}_{2}^{-}$ that lead to the highest concentration of SEG have a density between 1.4 and 1.5 g.cm^−3^. ILs with densities around 1.4 g.cm^−3^ seem to be favorable to suspend exfoliated graphite, this conclusion being a simple observation and does not necessarily imply causation.

The same observations hold for the surface tension. Having an IL with a surface tension around 34 mN.m^−1^ leads to higher concentrations of SEG. In a previous work (Bordes et al., 2017), the interfacial energy between graphite and different ILs was calculated. On Figure 2C, we could not correlate this interfacial energy with the concentration of SEG in the studied ILs, because as it was discussed in a previous work (Bordes et al., 2017), for four ILs, the graphite-ILs interfacial energy is not a value but instead just an upper bound. We also investigated the speed of sound in four ILs, in order to correlate a quantity related to compressibility and cavitation with the concentration of SEG. We could not find a valuable trend because of the lack of values in literature (the results are presented in Figure S2). Coleman et al. (Hernandez et al., 2008) studied forty molecular solvents and predicted that good solvents are characterized by surface tensions in the region of 40–50 mN.m^−1^. In the case of ILs, surface tensions between 30 and 40 mN.m^−1^ lead to better stabilization of exfoliated graphite.

### 3.2. Lateral Size of Flakes

**Figure 3:**
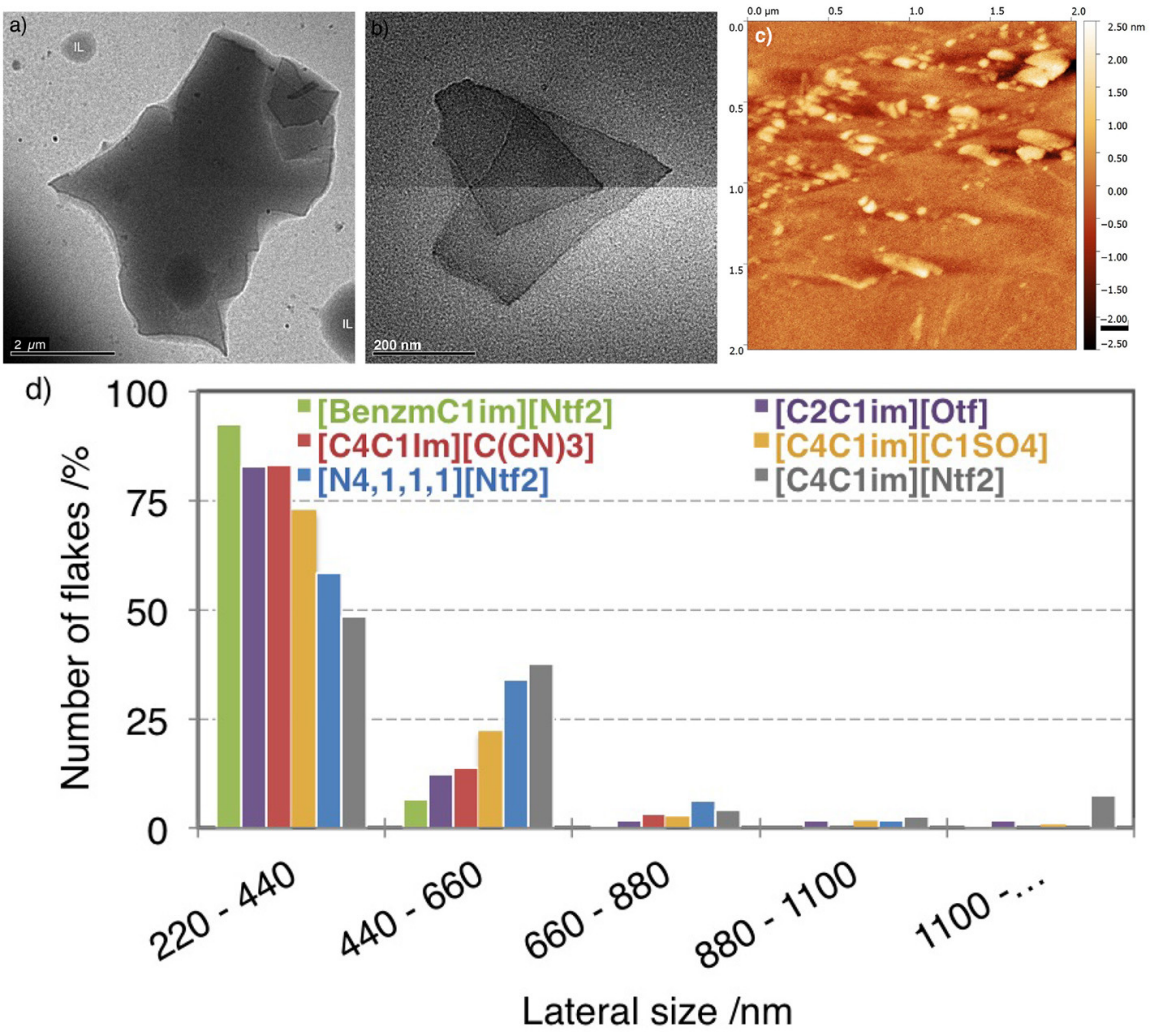
TEM images of multilayer graphene after centrifugation in **(a)** [C_4_C_1_im][Ntf_2_] and **(b)** [C_2_C_1_im][Otf]. **(c)** Topographical AFM image of flakes produced in [C_2_C_1_im][Otf] and deposited on Si/SiO_2_ substrates by spin-coating. **(d)** Histogram of the percentage of flakes found in the dispersions as a function of the lateral size of flakes.

We measured the lateral size of SEG in ILs using TEM and AFM. Figures 3a–c displays examples of flakes. At least fifty flakes of SEG, were analyzed to generate statistical results that are presented in the histogram of the Figure 3d. With our exfoliation process (24 h sonication plus centrifugation at 10,000 rpm) most of the SEG have sizes between 220 and 440 nm. Our results reveal that the lateral size of flakes exfoliated is larger in [C_4_C_1_im][Ntf_2_] than in [BnzmC_1_im][Ntf_2_].

Flakes of exfoliated graphite have larger sizes in [C_4_C_1_im][Ntf_2_] than in [N_4, 1, 1, 1_][Ntf_2_], and also appear to be present in suspension in larger quantity, in agreement with the measurement of concentration determined by absorption spectroscopy (Figure 1). The ionic liquids [C_2_C_1_im][Otf] and [C_4_C_1_im][C(CN)_3_] lead to similar lateral size profiles and to approximately the same amounts of suspended material. We conclude that ILs with the ${\text{Ntf}}_{2}^{-}$ anion favor the stabilization of larger exfoliated graphite flakes.

### 3.3. Number of Graphene Layers

**Figure 4:**
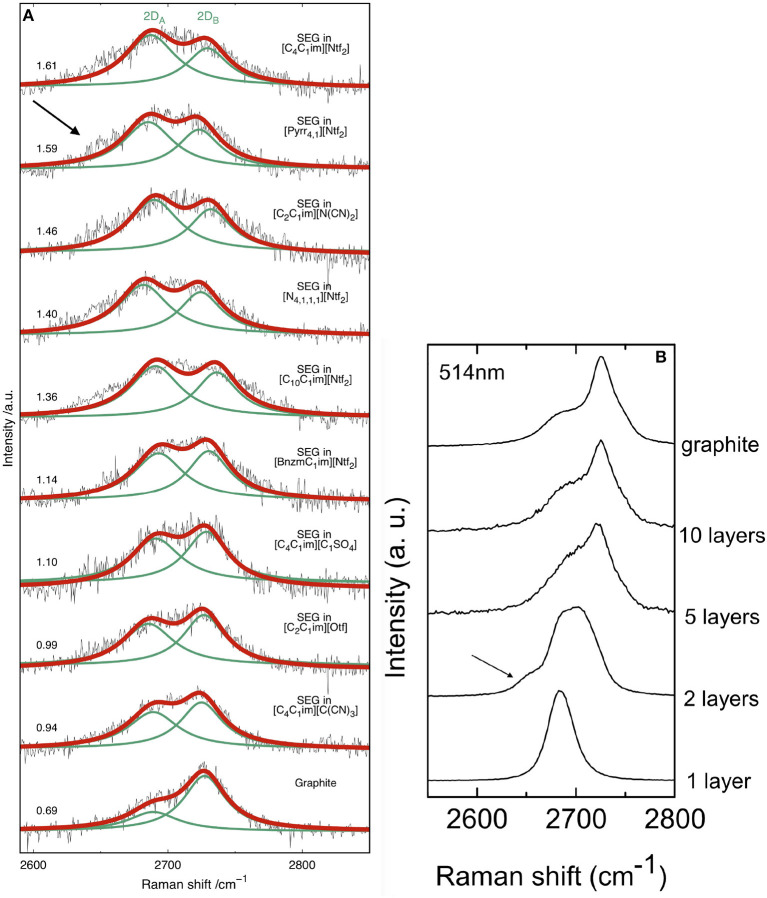
**(A)** Raman spectra (2D band) using a 514.5 nm laser for bulk graphite and suspended exfoliated graphite in different ionic liquids after filtration on a PVDF membrane. The values on the left correspond to the ratio of the areas of the two Lorentzian functions used to fit the 2D band. **(B)** Evolution of the 2D band with the number of graphene layers (514.5 nm). Reproduced with permission from Ferrari et al. (2006). Arrows indicate a shoulder present in the case of bilayer graphene.

In order to determine the number of layers in the exfoliated flakes we measured their Raman spectra in nine ILs and compared those with the spectrum of bulk graphite (Figure 4). The flakes of SEG were obtained by filtering the suspensions though a PVDF membrane, but even after washing the filter (with dichloromethane and isopropanol) residual amounts of IL were found and caused some fluorescence during the acquisition of spectra.

The Raman spectra of graphene or graphite is mainly composed of D, G and 2D peaks corresponding to in-plane vibration (Ferrari and Basko, 2013). The G band appearing at 1582 cm^−1^ is assigned to the stretching of the C-C bond in graphitic materials. The D band (around 1350 cm^−1^) is due to the breathing modes of the carbon atoms and requires a defect, such as oxydation, to be activated. The intense 2D band at about 2,700 cm^−1^ is one of the D-band overtones characteristic of sp^2^ carbon materials (Malard et al., 2009). The Raman spectrum of graphite is given in the (Figure S1B).

Due to residual ionic liquid at the surface of graphite, inducing extra fluorescence, the resolution of the spectra is not sufficient to characterize properly the intensity of the D peak. In these conditions, the most relevant information obtained from the spectra is the shape of the 2D band. This is the reason why the relative 2D/D intensity is not described in this study. Accumulation of spectra between 2,600 and 2,850 cm^−1^ (corresponding to the 2D band) enabled us to reach a sufficient resolution for our analysis. The exact number of layers was measured using AFM and the presence of impurities was detected using XPS, as reported below.

We will focus on the shape on the 2D band, which changes significantly in shape and intensity when moving from graphene to graphite (Ferrari et al., 2006). Depending on the number of graphene layers, the 2D band can be fitted with a different number of Lorentzian function (Ferrari et al., 2006; Zhu et al., 2014). Comparing our spectra of Figure 4A with those of the literature (Figure 4B), we can see our samples are not monolayer graphene because the bands are too broad.

According to the literature (Malard et al., 2009), the number of Lorentzian functions needed to fit the 2D peak is linked to the number of graphene layers, although not in a monotonous way. In order to establish a direct comparison between the SEG in the nine ILs, we chose to fit the band with two Lorentzian functions, called 2D_A_ and 2D_B_, as our Raman spectra are too noisy to fit accurately with more functions. The half-width at half-maximum (hwhm) of 2D_A_ and 2D_B_ have been kept fixed for every spectrum. The ratio of the areas of the two Lorentzian functions used for fitting this band are given in the Figure 4 with each spectrum. Raman spectra have been recorded at three different positions for each sample. The spectrum with the highest area 2D_A_/2D_B_ ratio is given in the Figure 4. Where we see that a correlation exists between the number of layers of graphene and the area ratio. In graphite, this ratio has a value of 0.69. For SEG obtained in the five ILs with an area ratio above 1.14, we can observe an shoulder on the left of the band at 2650 cm^−1^. This does not correspond to 5–10 graphene layers because the ratio of the two band is to high. The 2D band of the Raman spectra of SEG in [C_4_C_1_im][Ntf_2_], [Pyrr_4, 1_][Ntf_2_], [C_2_C_1_im][N(CN)_2_], [N_4, 1, 1, 1_][Ntf_2_] and [C_10_C_1_im][Ntf_2_] are similar to that of bi-layer graphene, as we can observe a shoulder in Figure 4.

**Figure 5:**
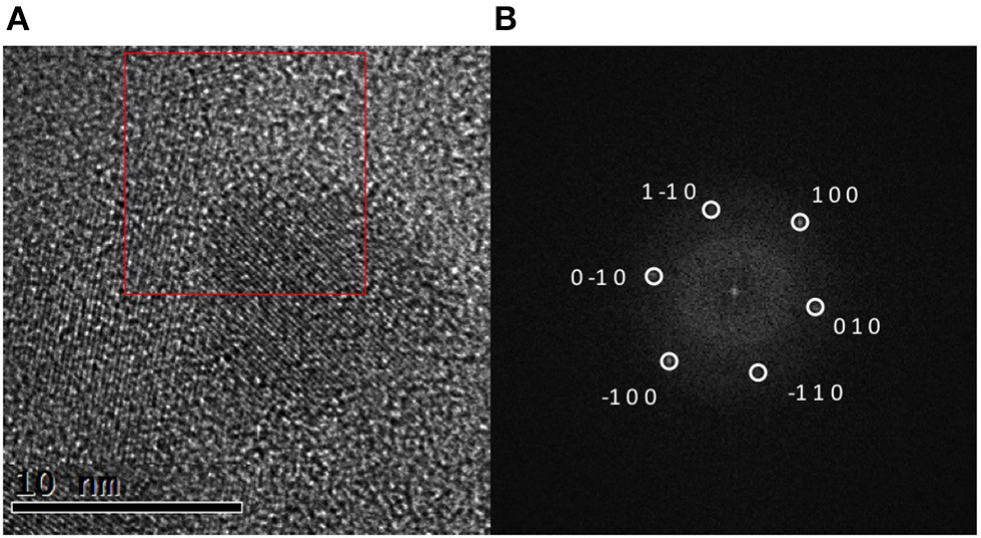
**(A)** Exemplary HRTEM image of graphene generated by exfoliation of graphite by sonication in [C_4_C_1_im][Ntf_2_] and **(B)** FFT of the area in the red square indexed by a hexagonal structure along the (001) zone axis with a lattice parameter of 2.5 and 6.7 Å corresponding to graphene.

The as-prepared graphene sheets were analyzed by high resolution HRTEM. The pictures contrast allowed an easy recognition of single to very few layered graphene from residual graphite existing in the sample. Additionally, graphene sheets exhibited a well-defined crystalline structure under HRTEM as shown in Figure 5A. As a result, fast-Fourier transform (FFT) analysis of the pictures yielded a pattern of clearly defined spots in the reciprocal space. This pattern was compared to the typical hexagonal close-packed (hcp) structure of graphene.

In all cases, spots corresponding to an interplanar distance of about 2.1 Å were observed. It could correspond to the (100) distance in the hcp structure. Indeed, it is shown in Figure 5B that the pattern in the FFT image can be completely indexed using this structure. The real electron diffraction pattern measured (in Figure S4) also supported the same crystal structure.

**Figure 6:**
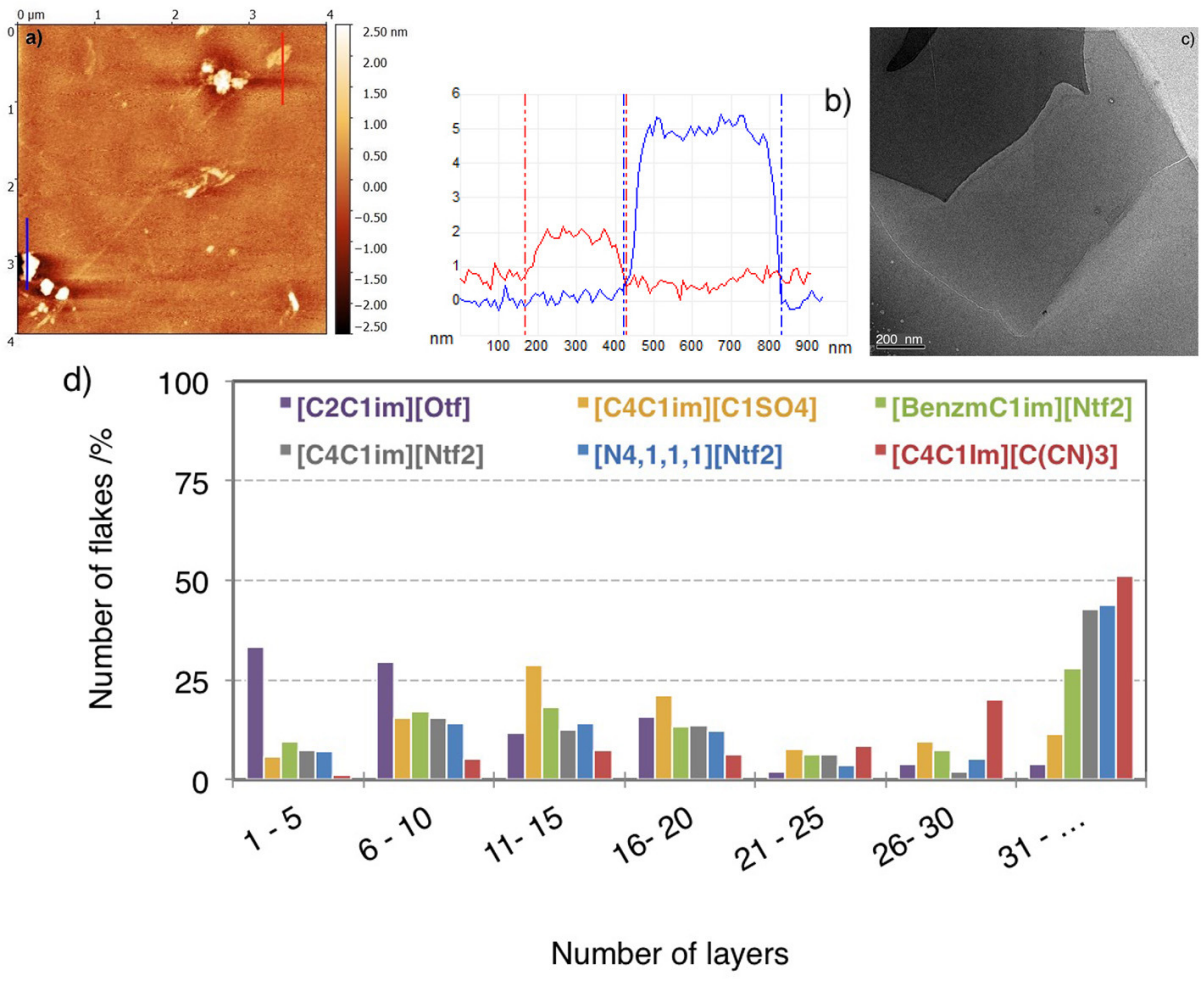
**(a)** Topographical AFM image of flakes produced in [C_2_C_1_im][Otf] and deposited on Si/SiO_2_ substrates by spin-coating. **(b)** Height profile of bi-layers graphene (red) and fourteen layers graphene (bleue). **(c)** TEM image of multilayer graphene in [C_4_C_1_im][Ntf_2_] **(d)** Histogram of the percentage of flakes as a function of the number of layers obtained statistical thickness analysis by AFM.

AFM is one of the most direct and precise methods to quantify the degree of exfoliation of graphene by measuring the height of the deposited flakes. Between 50 and 150 flakes were analyzed for each sample (Figures 6a,b). The superposition of layers illustrated in Figure 6c), would result in over-estimation of the number of graphene layers in AFM measurements. The estimation of the height of SEG *via* AFM depends on the substrate and on the experimental conditions (i.e., relative humidity and temperature). For example, on Si/SiO_2_, graphene can show an apparent height of 1 nm (Ishigami et al., 2007; Eredia et al., 2017). In this work, graphene height is estimated by assuming that the apparent thickness of the thinnest graphene sheet observed on our images amounts to 0.9 nm and the distance between two layers of graphene is 0.315 nm.

The SEG were also analyzed by high resolution TEM to identify monolayers of graphene. Figure 6d shows a histogram of the distribution of flake thickness for six samples. In all the liquids, graphene or few-layers graphene (≤ 5 layers) was found. A poorly exfoliated suspension was produced in [C_4_C_1_im][C(CN)_3_]. Fifty percent of the exfoliated graphene is in fact graphite with more than 30 flakes while in [C_2_C_1_im][Otf], 30% of graphene with <5 layers is found. Using Figure 6d a ranking can be estimated to find the IL, amongst those studied, which exfoliates the most of graphite: [C_2_C_1_im][Otf] > [C_4_C_1_im][C_1_SO_4_] > [BnzmC_1_im][Ntf_2_] > [N_4, 1, 1, 1_][Ntf_2_] ≃ [C_4_C_1_im][Ntf_2_] > [C_4_C_1_im][C(CN)_3_].

The choice of the anion seems important to control the degree of exfoliation of SEG. Adding a benzyl function instead of an alkyl chain on an imidazolium head group will reduce the lateral size of flakes but will favor a higher concentration of graphene.

### 3.4. Purity of Exfoliated Graphene

**Figure 7:**
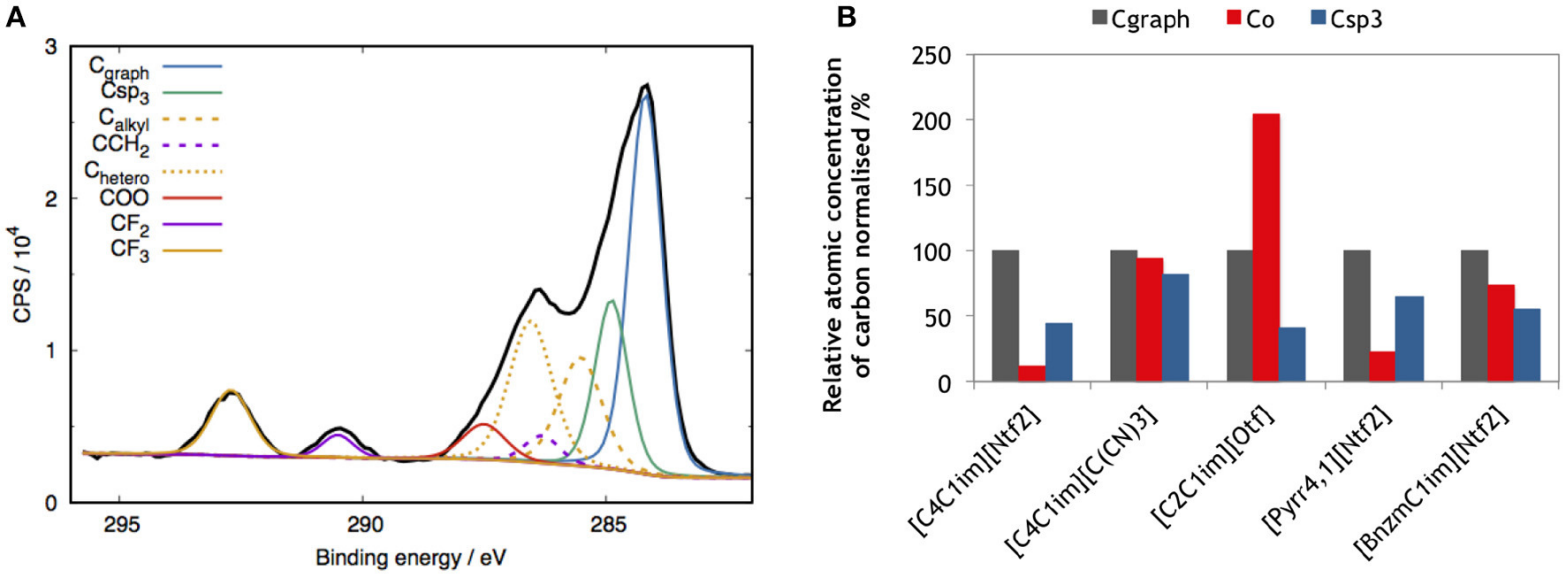
**(A)** XPS spectrum of SEG in [C_4_C_1_im][Ntf_2_] expressed in counts per second (CPS). Contributions of the deconvoluted carbon peaks: graphene/graphite; C_graph_ (blue curve), oxydized carbon; C_*O*_ (red curve), Csp_3_ (green curve), PVDF membrane (purple curves) and IL (orange curves). **(B)** Relative atomic concentration of carbon, normalized with respect to C_graph_, corresponding at the surface of exfoliated graphene in 5 ILs.

The purity of graphene after exfoliation in different ILs was investigated by XPS. In order to probe the relative amount of sp^2^, sp^3^ and oxydated carbon atoms at the surface of exfoliated graphite, XPS measurements were performed in samples of washed SEG deposited on a PVDF membrane. Based on references samples studies, we performed C1s core level fitting for exfoliated graphite in different ILs media. In exfoliated graphite, we assigned five main contributions, graphitic carbon (CC_graph_) at 284.3 eV, IL related carbon (C_alkyl_, C_hetero_ and CF_3_ at 285.6, 286.5 and 292.7 eV), PVDF related peaks (CCH_2_ and CF_2_ at 286.3 and 290.5 eV), carbon sp^3^ at 284.9 eV and carbon oxyde related peak (C–O and COO at 286.0 and 287.5 eV). Figure 7A shows peak fitting for SEG in [C_4_C_1_im][Ntf_2_]. The XPS spectrum of natural graphite is presented in the (Figure S5) for comparison. Since IL is present in relatively large quantities at the surface of graphene, the IL contributions were inferred from the pure IL XPS spectra. Hence the Csp^3^ contribution, where the binding energy is closed to alkyl and hetero carbon of ILs, was identified. Based on this peak fitting protocol, the relative concentrations of C_graph_, C–O and Csp^3^ are reported in the chart of Figure 7.

The purity of SEG in [C_4_C_1_im][Ntf_2_] and [Pyrr_4, 1_][Ntf_2_] is similar in terms of the presence of Csp^3^ and C–O at the surface. For the rest of the ILs studied, the oxydation at the surface is significant. Specially in the case of exfoliated graphite in [C_2_C_1_im][Otf]: more than two every three carbons are not graphitic. This result can be correlated with the number of graphene layers determined by AFM measurements in Figure 6d. [C_2_C_1_im][Otf] produced the most exfoliated graphite among the ILs we studied and it is known that one method to produce graphene form graphite is to oxidize the graphite (Zhu et al., 2010), so the high content of graphene in [C_2_C_1_im][Otf] could be due to oxydation at the surface of graphene. In the presence of [bnzmC_1_im][Ntf_2_] and [C_4_C_1_im][C(CN)_3_], formation of C-H bonds and oxidation of at least 60% of carbon were found at the surface of the carbon material.

The eventual degradation of pure IL due to sonication was investigated by XPS. A volume of 2 mL of [Pyrr_4, 1_][Ntf_2_] was sonicated during 24 h. No differences were found between the XPS spectra of the IL before and after sonication, as shown in the (Figure S6). This oxidation at the surface of graphene could be due to the presence of a few ppm of water in the ILs, to the gas dissolved in ILs or to the step of washing the filter with dichloromethane.

### 3.5. Free Energies of Peeling

**Figure 8:**
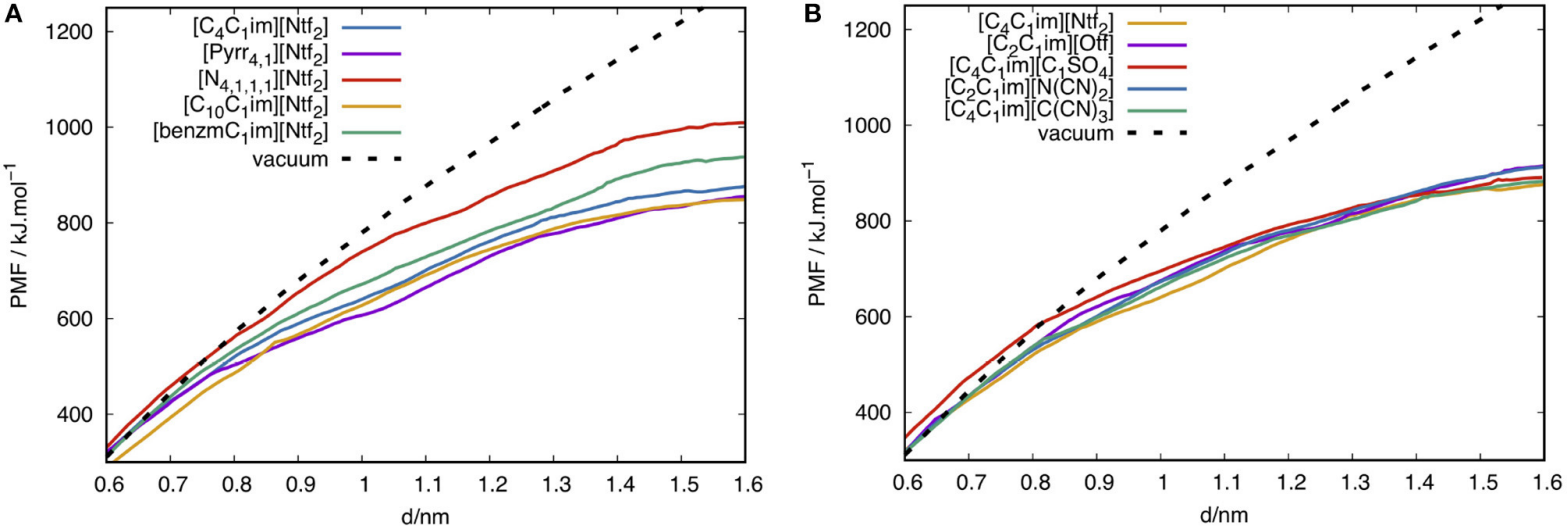
PMF of peeling one graphene layer from a stack of four layers in ILs at 423 K. Plot **(A)** corresponds to different cations and plot **(B)** to varying anions. d is the “vertical” separation between the edge of the single layer being peeled and the edge of the underlying layer.

In order to quantify the energy needed to exfoliate one graphene layer in the nine ILs, the reversible work (PMF) of peeling graphene in ILs was calculated from MD simulations and is plotted in Figure 8. The reversible work required to exfoliate graphene is higher in vacuum than in any of the ILs at 423 K. Thus, ILs are favorable solvents since they reduce the free energy for the exfoliation of graphene. Some results have been discussed in a previous work (Bordes et al., 2018).

In Figure 8, PMF curves obtained in different anions are closer than those obtained in different cations. Changing the anion leads to PMF curves that are too close to be completely distinguishable in our simulations, whereas different cations lead to PMF curves which are clearly distinguishable. The choice of the cation appears therefore more significant. The presence of a benzyl group is not beneficial toward the graphene peeling process, a conclusion in line with the absorbance measurements reported above (see Figure 1). Increasing the alkyl chain of the imidazolium cation decreases the free energy of peeling. The pyrrolidinium head group leads to an improvement in solvent quality compared to imidazolium and also to ammonium. This result was also expected from the concentrations of SEG in ILs. However, since the imidazolium head group is aromatic we expected stronger interactions with graphene for imidazolium ILs. [N_4, 1, 1, 1_][Ntf_2_] has demonstrated worst results than [C_4_C_1_im][Ntf_2_], in term of concentration, lateral size and number of exfoliated layers.The PMF curves also indicate that the intercalation of [N_4, 1, 1, 1_][Ntf_2_] between two layers of graphene requires more energy than [C_4_C_1_im][Ntf_2_].

## 4. Conclusion

In order to attain a more complete view of suspended graphene in ILs, different techniques were used to characterize graphene flakes produced by solvent exfoliation of graphite.

The concentration of suspended exfoliated graphene (SEG) is related to the stabilization step. UV-visible spectroscopy measurements reveal that the ILs with ${\text{Ntf}}_{2}^{-}$ anions stabilize higher concentrations of exfoliated graphite. Higher concentrations of SEG are produced in ILs with medium-length alkyl side chains in cations and without functionalisation by benzyl groups. Density and surface tension of the ILs seem to be good descriptors to choose ionic liquids capable of stabilizing larger concentrations of SEG.

AFM and TEM measurements allowed to determine the lateral size of graphene flakes, showing that [C_4_C_1_im][Ntf_2_] and [N_4, 1, 1, 1_][Ntf_2_] lead to larger flakes compared to [BnzmC_1_im][Ntf_2_] whereas smaller SEG flakes were found in [C_2_C_1_im][Otf]. A bulky anion seems to be an advantage to stabilize larger flakes. The number of stacked layers in flakes determined by AFM was smaller in [C_2_C_1_im][Otf].

Exfoliation of graphene in ILs using ultrasound caused surface changes mainly oxydation but also sp^3^ hybridization of carbon. [C_2_C_1_im][Otf] leads to the highest degree of oxidation compared to the others ILs studied. This result could explain the efficiency of this IL to exfoliate graphite down to single layers. Using an ultrasonic bath to process the exfoliation of graphite does not seems to be the best technique to produce a high concentration of pristine graphene. To optimize the exfoliation process, we need first to increase the reproducibility of the sonication/centrifugation procedure used in this work in order to increase the initial concentration and size of graphite flakes significantly.

Adding π-π interactions between the IL and the carbon material does not seem to improve the exfoliation process. With these results, the design of the IL can be optimized by choosing a ${\text{Ntf}}_{2}^{-}$ anion to increase the stabilization and the purity of SEG with a 1-ethyl-2-methylpyrrolidinium cation to increase the intercalation and the dispersion of graphene flakes.

## Author Contributions

EB conducted the experiments jointly with BM (TEM), CS (TEM and EIS), DB and P-OB (AMF), AB (XPS, EIS). MG and AP supervised the project. The manuscript was written by EB, MG, and AP with inputs from all authors.

### Conflict of Interest Statement

The authors declare that the research was conducted in the absence of any commercial or financial relationships that could be construed as a potential conflict of interest.
