# Corrigendum: Dispersion and Stabilization of Exfoliated Graphene in Ionic Liquids

**DOI:** 10.3389/fchem.2020.00556

**Published:** 2020-07-03

**Authors:** Emilie Bordes, Bishoy Morcos, David Bourgogne, Jean-Michel Andanson, Pierre-Olivier Bussière, Catherine C. Santini, Anass Benayad, Margarida Costa Gomes, Agílio A. H. Pádua

**Affiliations:** ^1^Centre National de la Recherche Scientifique, SIGMA Clermont, Institut de Chimie de Clermont-Ferrand, Université Clermont Auvergne, Clermont-Ferrand, France; ^2^UMR 5265 Centre National de la Recherche Scientifique, Université de Lyon, Villeurbanne, France; ^3^Chemistry Department, Faculty of Science, Alexandria University, Alexandria, Egypt; ^4^Université Grenoble Alpes and CEA, LITEN, Grenoble, France; ^5^École Normale Supérieure de Lyon, Centre National de la Recherche Scientifique, Laboratoire de Chimie, Lyon, France

**Keywords:** exfoliation, graphene, graphite, ionic liquids, suspension

In the published article, there was an error regarding the affiliation for Bishoy Morcos. As well as having affiliation 2, they should also have “Chemistry Department, Faculty of Science, Alexandria University, Alexandria, Egypt” which has been added as affiliation 3.

The authors apologize for this error and state that this does not change the scientific conclusions of the article in any way. The original article has been updated.

