# Supplementary material for: Dispersion and Stabilization of Exfoliated Graphene in Ionic Liquids
Source: Front Chem. 2019 Apr 16;7:223. doi: 10.3389/fchem.2019.00223 (PMC6477887; doi:10.3389/fchem.2019.00223)
Supplement: Supplementary file 1 [file Data_Sheet_1.PDF]

Supplementary information
Dispersion and stabilisation of exfoliated graphene in ionic liquids

Emilie Bordes,[†] Bishoy Morcos,[‡] David Bourgogne,[†] Jean-Michel Andanson,[†] Pierre-Olivier Bussière,[†] Catherine C. Santini,[‡] Anass Benayad,[¶] Margarida Costa Gomes,[*,§] and Agílio A.H. Pádua[*,§]

†*Université Clermont Auvergne, CNRS, SIGMA Clermont, Institut de Chimie de Clermont-Ferrand, 63000 Clermont–Ferrand, France.*

‡*Université de Lyon, UMR 5265 CNRS, 43 bd du 11 Novembre 1918, 69616 Villeurbanne, France*

¶*Université Grenoble Alpes and CEA, LITEN, France*

§*École Normale Supérieure de Lyon, CNRS, Laboratoire de Chimie, 69364 Lyon, France*

E-mail: margarida.costa-gomes@ens-lyon.fr; agilio.padua@ens-lyon.fr

Water content in ionic liquids

After ionic liquids (ILs) have been dried under primary vacuum for 24 hours at room temperature, the water content has been quantified by a Karl Fisher coulometer DL32 from Metter Toledo in a Hydranal solution.

Table S1: Water content in different liquids used to exfoliate the graphite.

Formula	Name	Water content /ppm
$[C_4C_1im][Ntf_2]$	1-butyl-3-methylimidazolium bis(trifluoromethylsulfonyl)imide	10-180
$[C_{10}C_1im][Ntf_2]$	1-decyl-3-methylimidazolium bis(trifluoromethylsulfonyl)imide	210
$[BnzmC_1im][Ntf_2]$	1-benzyl-3-methylimidazolium bis(trifluoromethylsulfonyl)imide	480
$[Pyrr_{4,1}][Ntf_2]$	butylmethylpyrrolidinium bis(trifluoromethylsulfonyl)imide	20
$[N_{4,1,1,1}][Ntf_2]$	butyltrimethylammonium bis(trifluoromethylsulfonyl)imide	10
$[C_4C_1im][C(CN)_3]$	1-butyl-3-methylimidazolium tricyanomethanide	50
$[C_2C_1im][N(CN)_2]$	1-ethyl-3-methylimidazolium dicyanamide	270
$[C_4C_1im][C_1SO_4]$	1-butyl-3-methylimidazolium methylsulfate	160
$[C_2C_1im][Otf]$	1-ethyl-3-methylimidazolium triflate	60

Characterization of natural graphite flakes

Natural flakes graphite were purchased from Alpha Aesar with a 99.8% purity and a size inferior to 325 mesh. Raman spectroscopy and X-ray diffraction (XRD) were performed, without any treatment, to ensure the initial purity.

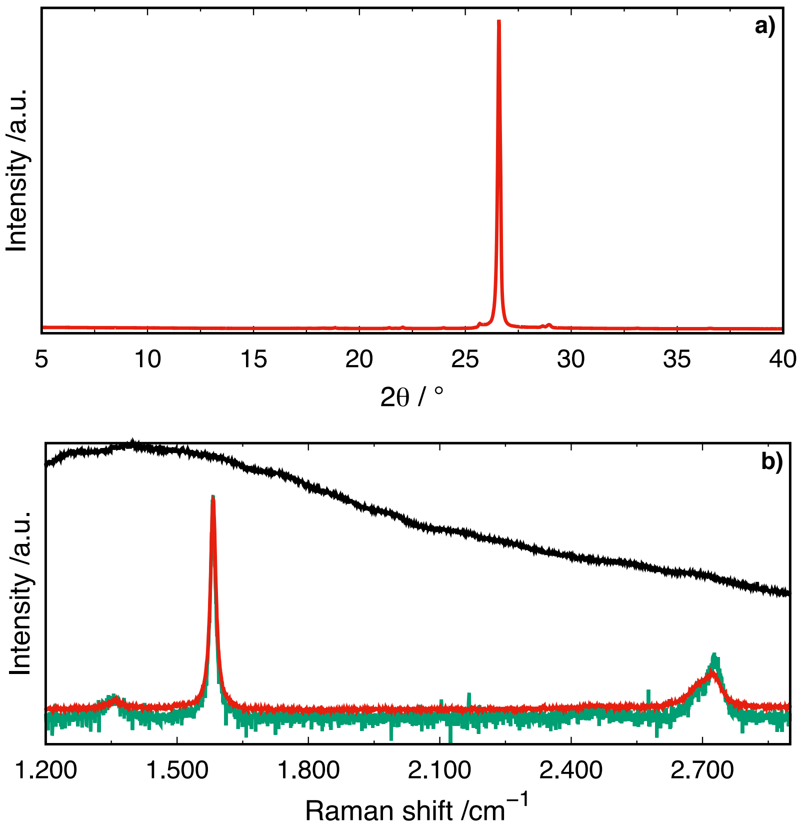

Figure S1: Characterization of graphite flakes from Alfa Aesar without specific conditioning: a) XRD and b) Spectroscopy Raman: graphite flakes (red), graphite flakes on PVDF filter (green) and PVDF filter (black).

Concentration of SEG in ILs

Table S2: Quantities of suspended exfoliated graphite (SEG) in ILs after centrifugation determined by spectroscopy UV-visble at 660 nm, at 298 K. Size of graphite flakes has been classified after filtration using a PVDF filter with pore size of 220 nm.

ILs	Yield of SEG	Concentration	Yield of SEG > 220 nm
Units	%	$mg.mL^{-1}$	%
$[C_4C_1im][Ntf_2]$	31.0	1.55	51.3
$[C_{10}C_1im][Ntf_2]$	20.4	1.01	52.9
$[BnzmC_1im][Ntf_2]$	20.9	1.08	70.7
$[N_{4,1,1,1}][Ntf_2]$	25.7	1.36	84.2
$[Pyrr_{4,1}][Ntf_2]$	35.0	1.73	59.4
$[C_2C_1im][Otf]$	3.4	0.18	44.3
$[C_4C_1im][C_1SO_4]$	9.1	0.47	85.0
$[C_4C_1im][C(CN)_3]$	1.5	0.08	100.0
$[C_2C_1im][N(CN)_2]$	4.3	0.22	31.6

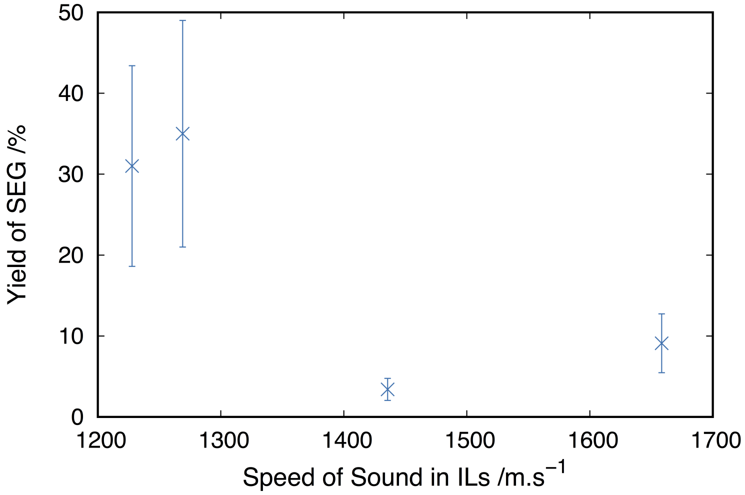

Figure S2: Yield of SEG in ILs as function of the speed of sound in ILs at 298 K.[1]

Size analysis of SEG in ILs

Table S3: Number of exfoliated graphene flakes analysed by AFM and TEM measurements in the different ILs.

ILs	Number of AFM	analysed flakes TEM	Average of number of layers (AFM)
$[C_4C_1im][Ntf_2]$	96	27	44.9 ± 38.4
$[C_{10}C_1Im][Ntf_2]$	12	13	
$[BnzmC_1im][Ntf_2]$	187	16	25.3 ± 17.3
$[N_{4,1,1,1}][Ntf_2]$	57	21	41.2 ± 32.1
$[Pyrr_{4,1}][Ntf_2]$	12	10	
$[C_2C_1im][Otf]$	51	10	11.5 ± 7.7
$[C_4C_1im][C_1SO_4]$	104	15	19.7 ± 9.8
$[C_4C_1im][C(CN)_3]$	94	3	38.8 ± 18.5
$[C_2C_1im][N(CN)_2]$	13	8	

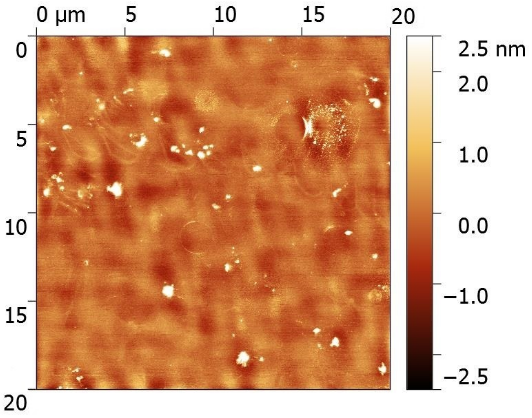

Figure S3: Topographical AFM image of flakes produced in IL and deposited on Si/SiO_2 substrates by spin-coating.

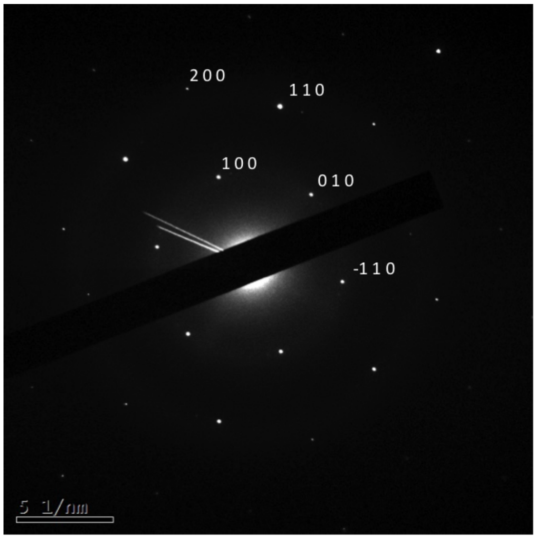

Figure S4: Electron diffraction pattern of graphene generated by exfoliation of graphite by sonication in [BnzmC_1im][Ntf_2].

XPS

The pristine graphite was fitting with an asymmetric peak centered at 284.5 eV and a plasmonic peak at 6.1 eV from the main peak relative to $\pi - \pi^*$ interaction. Spectrum is presented in Fig. S6.

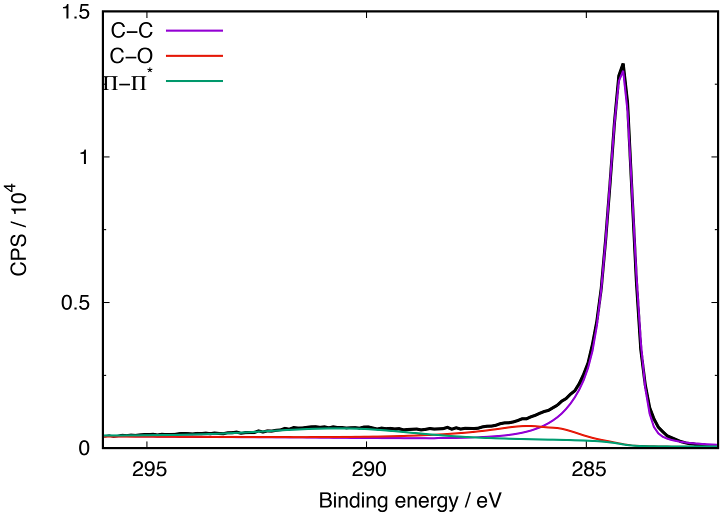

Figure S5: XPS spectrum, expressed in counts per second (CPS), measured at graphite surface. The deconvoluted carbon peak is presented.

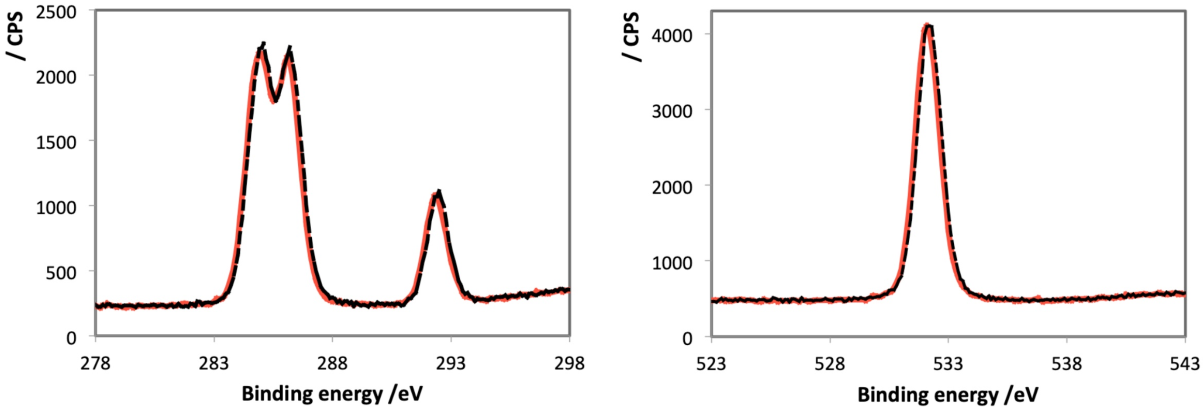

Figure S6: High resolution XPS spectrum, expressed in counts per second (CPS), measured on [Pyrr$_{4,1}$][Ntf$_2$] before (black dashed line) and after (red line) sonication for 24h at 423 K. The peaks presented are for C (left) and O (right).

References

(1) Sattari, M.; Gharagheizi, F.; Ilani-Kashkouli, P.; Mohammadi, A. H.; Ramjugernath, D. Determination of the speed of sound in ionic liquids using a least squares support vector machine group contribution method. *Fluid Phase Equilib.* **2014**, *367*, 188–193.
